# Spot the Pneumothorax

**DOI:** 10.7759/cureus.17177

**Published:** 2021-08-14

**Authors:** Joshua Harridine, Milind Sovani

**Affiliations:** 1 Acute Respiratory Care Unit, Nottingham University Hospital, Nottingham, GBR; 2 Respiratory Medicine, Nottingham University Hospital, Nottingham, GBR

**Keywords:** secondary pneumothorax, surgical emphysema, chest drain, subcutaneous drain, acute medical emergencies management

## Abstract

Subcutaneous emphysema (SE) and pneumomediastinum are commonly associated with critically ill patients with blunt or penetrating trauma, in particular lower rib fractures. It however rarely needs urgent intervention, and routine use of chest tube tracheostomy or mediastinal drains is not recommended as the patients do not go on to develop a respiratory compromise. Our case is novel as it describes a case of subcutaneous emphysema with acute upper airway compromise and respiratory distress requiring urgent bilateral wide bore subcutaneous drains and thoracic drain insertion. The patient required a prolonged recovery period.

This case serves to illustrate the technical difficulty in establishing a cause of subcutaneous emphysema, the limitations of standard imaging in identifying a pneumothorax in subcutaneous emphysema, and the value of prompt insertion of bilateral subcutaneous wide bore drains to buy precious time for definitive imaging and management.

## Introduction

Subcutaneous emphysema (SE) is commonly caused by blunt or penetrating chest trauma of which lower rib fractures are a major risk factor. Extensive SE left untreated in the presence of dysphagia, dysphonia, and airway compromise causing the “tension phenomenon” [1] is life-threatening and requires urgent treatment.

## Case presentation

A 75-year-old female attended the accident and emergency department with increased work of breathing and shortness of breath due to an exacerbation of chronic obstructive pulmonary disease (COPD). She had a background of ischaemic heart disease (IHD), previous bilateral pulmonary emboli (PE) with long-term anticoagulation, hypertension, COPD, bilateral hip replacements, and a recent admission to hospital with a suspected Mallory-Weis Tear. Initially, observations were within normal confines; a normal chest X-ray (CXR) was noted and blood reports were unremarkable, other than hyponatraemia of 124 mmol/L and hypercapnia on her arterial blood gas. Due to her increasing hypercapnia and borderline acidosis, she was transferred to a level 1 bed in the acute respiratory care unit (ARCU). It was decided that her ceiling of care was ARCU and she would not be referred to intensive care if she became more unwell.

During the evening of day three of admission, the patient had a fall onto her right side and suffered a blow to her head. At the time, her observations showed that she had a blood pressure of 86/40 mm/Hg that likely contributed to the fall. She had a reduced Glasgow Coma Score (GCS) of 14/15 due to confusion and she complained of right flank tenderness. Since she was anticoagulated, the on-call doctor arranged a CT head, which showed a right orbital haematoma but was otherwise within the normal confines for this patient’s age.

The following morning, during the ward round, the patient was noted to be more unwell than the previous day, talking in one-word answers and complaining of pleuritic chest pain. It was noted that her voice had altered, sounding more strangled.

The patient became increasingly tachycardic and the medical team was asked to review. Over the course of a few minutes, the patient developed extensive swelling across the whole upper body, abdomen, and face to the extent of which she was unable to open her eyes. On palpation of the swelling, crepitus was felt.

On assessment, the patient's airway was patent with a difficult-to-hear voice and central trachea. On auscultation, there was bilateral air entry and clinically no obvious pneumothorax. She reported tenderness over the right chest wall at ribs 4-6. The respiratory rate was 30 and saturation was 89% on air. There were no signs of circulatory compromise with a blood pressure of 107/64 mm/Hg and a heart rate of 140 beats per min. The GCS was 15/15 with blood sugar within the normal range.

**Figure 1:**
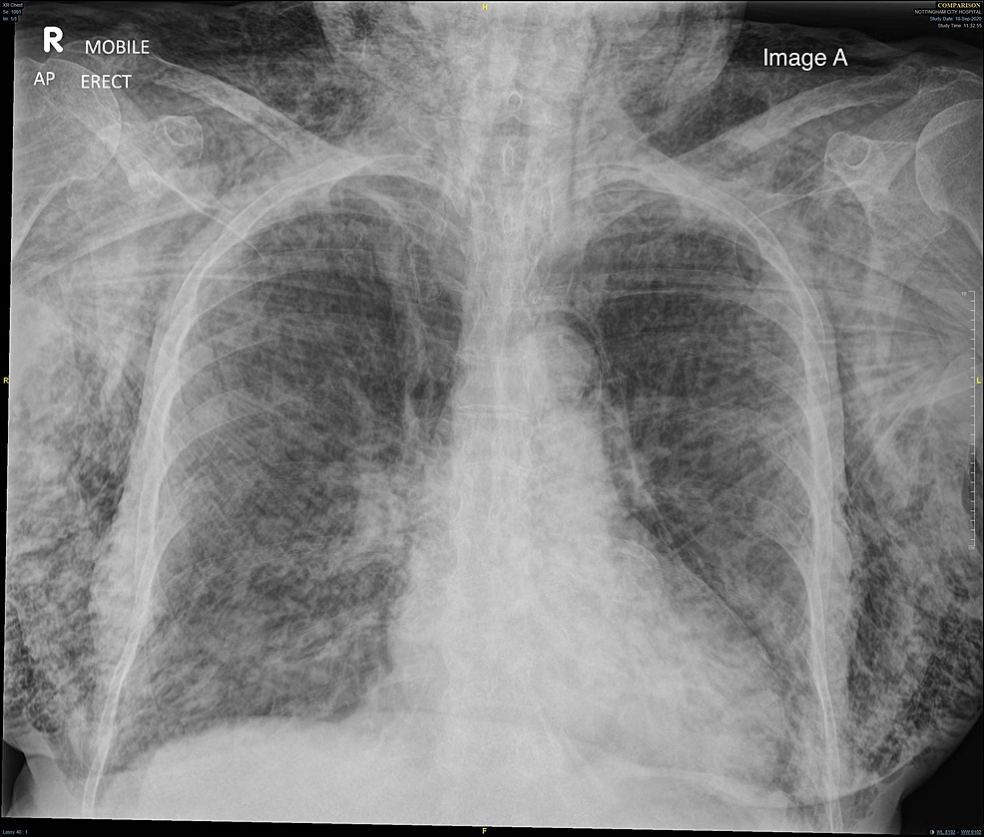
Subcutaneous emphysema chest X-ray

It was decided that the palpable crepitus felt was likely subcutaneous emphysema and that there was an air leak in the thoracic cavity. An urgent portable CXR (Figure 1) was requested and this showed extensive SE and pneumomediastinum. The patient deteriorated and developed signs of airway compromise with stridor becoming prominent.

An urgent thoracic surgical review was requested and the on-call thoracic surgeon attended to place two surgical chest drains subcutaneously to decompress the SE, preceded by 10 ml of subcutaneous lidocaine 1% to each insertion site. This had an immediate effect in reducing the respiratory rate and alleviating stridor.

**Figure 2:**
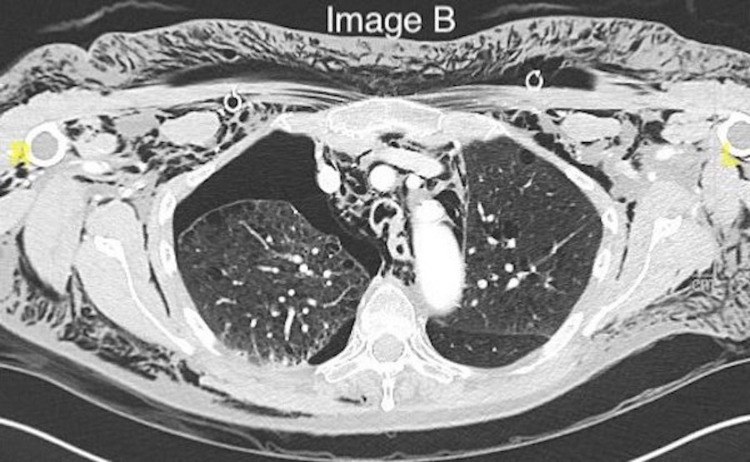
CT thorax demonstrating extensive surgical emphysema, bilateral subcutaneous chest drains, and a right-sided pneumothorax

At this time, the cause of SE was unclear. A CT contrast of the thorax (Figure 2) was chosen to investigate the cause of SE. The CT showed a large right-sided pneumothorax with extensive subcutaneous emphysema coupled with 1st and 2nd rib fractures on the right.

The patient returned to the ward where she had a right-sided chest drain sited to resolve the pneumothorax. It was unclear if the oesophagus had perforated from the CT scan and there was a concern that this may have been a possible cause of the pneumomediastinum, and as a result, the patient remained nil by mouth and a contrast swallow study was arranged.

The contrast swallow was performed and it showed no obvious perforation in the oesophagus. The patient was recommenced on her regular diet. The chest drain was removed following the resolution of the pneumothorax, confirmed radiologically and after a prolonged stay in a level-1 bed was able to step down to the wards and eventually home.

## Discussion

This patient was an older adult who developed acute respiratory compromise and stridor from a rib fracture causing pneumothorax, pneumomediastinum, and subcutaneous emphysema.

Subcutaneous emphysema is an accumulation of subcutaneous air in multiple tissue planes. In extreme cases, it can cause airway compromise, cardiac tamponade, and tension pneumomediastinum [2]. With this patient, the issue was an accumulation of air in the deeper fascial planes leading to airway compromise; Beg et al. also note the risk of compression to the venous return and the blood flow to the head and neck [3]. Management focuses on protecting the airway while also encouraging the drainage of the SE.

Aghajanzadeh et al. [4] explored a classification system for the SE with grades 1-5: grade 1 including the base of the neck, grade 2 including the whole neck, grade 3 including subpectoralis area, grade 4 including the chest wall and all of the neck area, and grade 5 including the chest wall, abdominal wall, neck, orbits, scalp, and upper limbs. This patient would have been classified as grade 5 owing to the extensive SE she experienced; the same article identified four main causes of SE in their patient cohort as pneumothorax with a background of COPD in 34% of cases and trauma due to rib fracture being the second most common with 31% of cases as well as iatrogenic damage in 26% and barotrauma in 9% of cases. This patient had a pneumothorax on a background of COPD and had experienced a rib fracture while an inpatient making her extremely high-risk for the development of SE.

There are multiple different techniques revealed by the literature search used to treat SE ranging from infraclavicular incisions [5], catheter insertion, insertion of chest tubes intrapleural, or subcutaneously [6] with or without suction [7]. However, the majority of previous treatment of SE focused on tracheostomy because it effectively and rapidly establishes an adequate airway, keeps the deeper tissue planes in the thoracic inlet and the neck decompressed, and maintains positive intrathoracic pressure allowing the lungs to fully expand. Insertion of a tracheostomy is a challenging procedure not often used outside of anaesthetics and critical care was not deemed an option for this patient; however, subcutaneous drains provided a simpler, cheaper, and less labour intensive method of management with similar efficacy in the resolution of SE [8].

Insertion of 14 g angiocatheter has the advantage of being minimally invasive and simple to do, and it is well-tolerated due to the insensitivity caused by the massive distension [9]. However, the small size of a catheter means it has the potential to become occluded by blood and discharge, rendering it useless. A large-bore subcutaneous drain has the advantage of being significantly larger so less likely to become blocked; however, its size does necessitate a specialist for insertion and local anaesthesia, which can potentially delay treatment of SE. 

## Conclusions

Subcutaneous emphysema developing with acute respiratory compromise following a traumatic fall is associated with large pneumothoraxes, which can be impossible to identify with standard imaging modalities and require cross-sectional imaging (CT scan). In situations complicated by signs of impending airway compromise, the insertion of bilateral subcutaneous drains should be performed as a time-buying measure.
